# Association between an ITHBC theory-based nursing pathway and discharge readiness among mothers of preterm infants: a non-randomized controlled study with a historical control group

**DOI:** 10.3389/fmed.2026.1938054

**Published:** 2026-10-20

**Authors:** Fang Tang, Yihong Wu, Liping Dai, Wenbo Yan, Meng Yang, Weiyi Shi, Achao Tian, Yonghao Zhang

**Affiliations:** 1 Department of Nursing, Tongren Maternal and Child Health Care Hospital, Tongren, Guizhou, China; 2 Department of Neonatology, Tongren Maternal and Child Health Care Hospital, Tongren, Guizhou, China; 3 Department of Obstetrics, Tongren Maternal and Child Health Care Hospital, Tongren, Guizhou, China; 4 Department of Pediatric Rehabilitation, Tongren Maternal and Child Health Care Hospital, Tongren, Guizhou, China; 5 Psychological Clinic, Tongren Maternal and Child Health Care Hospital, Tongren, Guizhou, China

**Keywords:** Integrated Theory of Health Behavior Change, mother, nursing pathway, preterm infants, readiness for hospital discharge

## Abstract

**Objective:**

To evaluate the effects of an Integrated Theory of Health Behavior Change (ITHBC)-based nursing pathway on discharge readiness in mothers of preterm infants.

**Methods:**

In this non-randomized controlled study with a historical (temporal) control group, 115 mothers were divided into an observation group (ITHBC-based care, *n* = 57) and a control group (routine care, *n* = 58). Discharge readiness, discharge instruction quality, care ability, self-efficacy, resilience, anxiety, and depression were assessed at discharge; infant growth, complications, readmission, and maternal satisfaction were followed up at 6 months. Multivariable linear regression was used to adjust for baseline covariates for key outcomes.

**Results:**

The observation group showed significantly higher discharge readiness, discharge instruction quality, care ability, self-efficacy, and resilience, with lower anxiety and depression (all *P* < 0.05). At 6 months, their infants had better growth metrics, fewer complications (thrush, diaper rash), and lower readmission rates, alongside higher maternal satisfaction (*P* < 0.05). These differences remained significant after covariate adjustment.

**Conclusions:**

The ITHBC-based nursing pathway was associated with better maternal psychological status and care competence, more favorable infant health outcomes, lower readmission rates, and higher satisfaction, providing a practical framework for transitional care in preterm infants.

## 1 Introduction

Preterm birth is defined as birth before 37 completed weeks of gestation. It is one of the leading causes of death and disability among children under 5 years of age (1). The survival rate of preterm infants has greatly increased with advancements in perinatal medicine and the widespread use of neonatal intensive care technologies; however, the necessity of ongoing care following discharge has emerged (2). Preterm infants continue to face a number of health risks, including feeding difficulties, growth retardation, and a significant risk of infection after discharge, due to the immature development of many organs and systems. The long-term quality of life is directly correlated with the quality of care given to preterm newborns (3).

In the care system for preterm infants, mothers are the primary providers of continuous care. However, due to early mother–infant separation and a sudden role change, mothers of preterm infants generally experience anxiety and reduced self-efficacy (4). When a preterm infant meets the discharge criteria, the mother must undertake complex home care tasks within a short period, making her readiness for discharge—defined as her perceived ability and preparedness to provide post-discharge care—a critical factor influencing the infant's post-discharge health outcomes. A previous study has shown that mothers with insufficient discharge readiness are prone to care overload, which increases the risk of unplanned readmission for preterm infants (5). Mothers’ psychological states and the process of changing their behavior were not given adequate attention in previous discharge guidelines, which primarily focused on centralized teaching on the day of discharge. The Integrated Theory of Health Behavior Change (ITHBC) provides a theoretical foundation for optimizing this procedure. The theory highlights how various factors, including knowledge, belief, and self-efficacy, as well as the dynamic evolution of intention, behavior, and maintenance, influence the formation of healthy behavior (6). Interventions based on ITHBC, which emphasize phased assessment and targeted support, have achieved good results in chronic disease management; however, the application of ITHBC among mothers of preterm infants remains limited.

In this context, the purpose of this study is to investigate how nursing pathways based on ITHBC affect the discharge readiness of mothers of preterm infants. This study aims to provide a theoretical foundation and practical reference for developing a scientific and systematic transitional care model for preterm infants by implementing phased and individualized nursing interventions for their mothers, observing the effects on discharge readiness and anxiety levels. This may ultimately promote the healthy development of preterm infants and the physical and mental wellbeing of both mothers and infants.

## 2 Materials and methods

### 2.1 Study design and participants

This was a non-randomized controlled study with a historical (temporal) control group. The study participants were mothers of preterm infants admitted to the Tongren Maternal and Child Health Care Hospital's Department of Neonatology between August 2024 and September 2025. The inclusion criteria were as follows: (1) met the criteria for diagnosing preterm infants in the field of medicine for preterm infants; (2) had a single preterm infant with a stable condition, a gestational age of ≥28 weeks and <37 weeks, a weight of >1,500 g; (3) mothers of preterm infants had no hearing, intelligence or expression disorders; (4) mothers of preterm infants had basic reading and comprehension ability, and their clinical data were complete. The exclusion criteria were as follows: (1) preterm infants with severe congenital diseases requiring surgical treatment in the neonatal period; (2) women with major chronic illnesses or diseases affecting the heart, brain, lungs, kidneys, or other organs; (3) incomplete clinical data or loss of follow-up after discharge.

Because the ITHBC-based pathway was implemented as a unit-wide practice change from May 2025 onward, a concurrent control group was not feasible after implementation. Therefore, mothers admitted before implementation (August 2024–April 2025) served as a historical control group, while those admitted after implementation (May–September 2025) received the intervention. Initially, 78 mothers in the control period and 72 mothers in the observation period met the inclusion criteria. After excluding those with incomplete baseline data (12 in the control group and 10 in the observation group) and those lost to follow-up at 6 months (8 in the control group and 5 in the observation group), 58 and 57 mothers remained in the final analysis, respectively. Due to the systematically implemented nature of the intervention, no individual randomization or matching of patients was performed. All information was gathered from standard follow-up archives, nursing documentation, and the hospital's electronic medical record system, including baseline characteristics, in-hospital evaluations, and 6-month post-discharge outcomes. The unit of assignment was the individual mother–infant pair. No major protocol deviations were identified during the study period. No matching or other allocation-phase strategies were used to minimize bias due to non-randomization; however, multivariable adjustment was performed in the analysis phase to partially account for baseline differences between groups. This non-randomized, historically controlled study with retrospective data collection was approved by the ethics committee of the hospital, which granted a waiver of informed consent. The study complied with the ethical principles of the Declaration of Helsinki. Figure 1 depicts the patient selection procedure.

**Figure 1:**
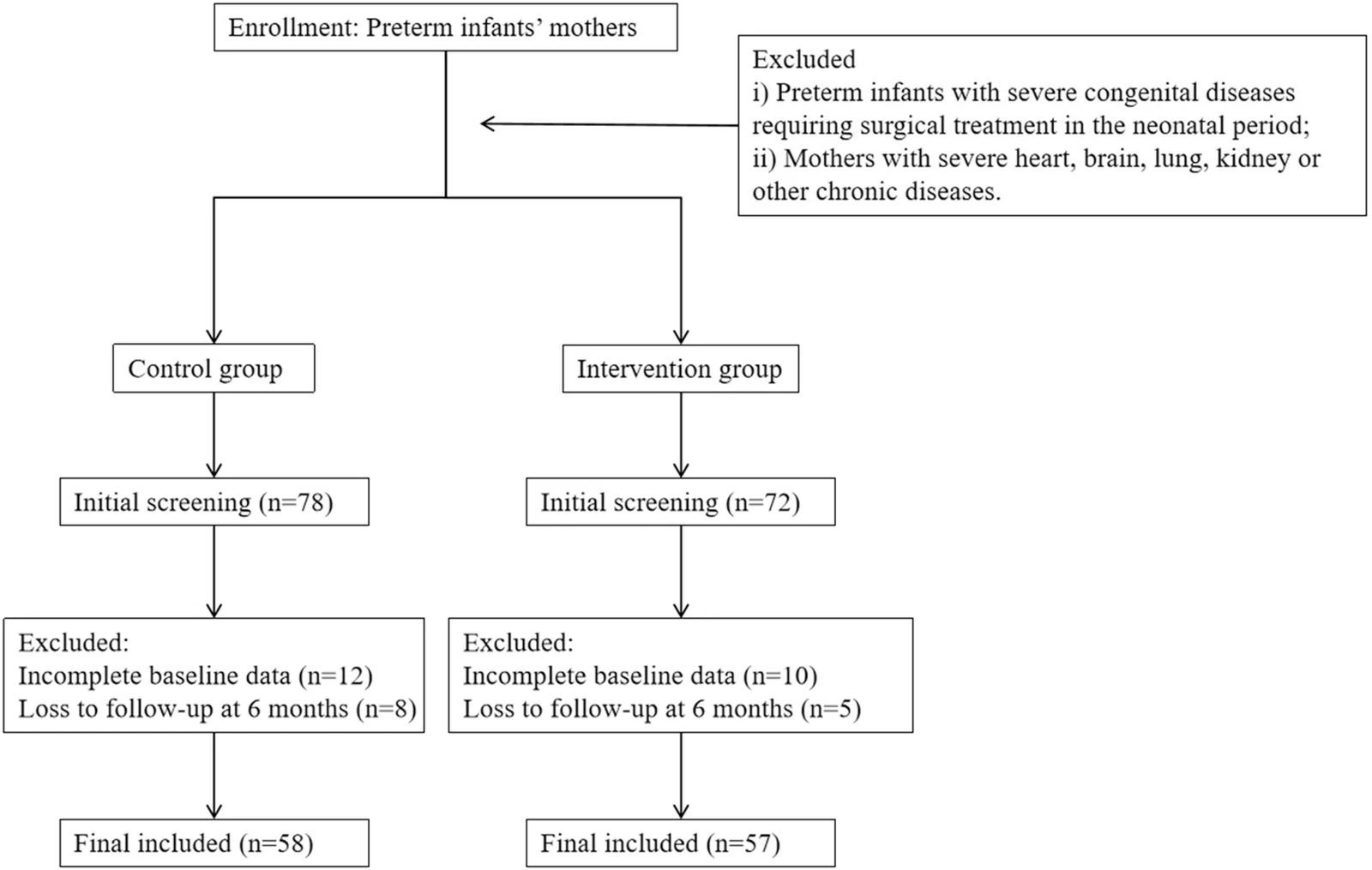
Flow diagram of participants.

### 2.2 Construction of nursing pathway based on ITHBC

#### 2.2.1 Establishment of the multidisciplinary team (MDT)

The MDT consisted of the head nurse with extensive clinical and scientific research experience, the supervisor nurse in the neonatology department, the attending physician in the neonatology department, the obstetrician-gynecologist, the rehabilitation physician, and the psychologist. The charge nurse oversaw organizing and executing the project, while the head nurse was responsible for its planning and execution. Other nurses and doctors collaborated in the creation of nursing strategies.

#### 2.2.2 Nursing strategy formulation based on ITHBC

MDT members carried out group discussions and summarized clinical experience from four aspects based on ITHBC: hospital health education, individual assessment and guidance, health planning and guidance, and creating a social support environment. Relevant clinical experience was summarized, and domestic and foreign literature on the discharge readiness of mothers of preterm infants was reviewed. The databases included PubMed, Embase, Web of Science, VIP, CNKI, and Wan Fang Data. Chinese search terms included: preterm infants, parturients, caregivers’ discharge readiness, education, rehabilitation; English search terms included: preterm infants, parturients, caregivers’ discharge readiness, education, rehabilitation. The search period was from 1 January 2010 to 1 July 2023. Based on the evidence-based and ITHBC, a preliminary plan was formed, including hospital health education, individualized assessment and guidance, health planning and guidance, and creating a social support environment. Based on this, expert consultation was carried out.

#### 2.2.3 Expert letter consultation

A total of 18 experts were invited for two rounds of Delphi expert letter consultation. The inclusion criteria were as follows: (1) worked in related fields for more than 10 years; (2) held an intermediate or above technical title, or a master’s degree or above; (3) familiar with preterm discharge readiness or health behavior theory; (4) voluntarily participated in and completed two rounds of letter consultation. The average age of the experts was 45.12 ± 6.84 years, ranging from 35 to 58 years. The average number of years of experience were 23.58 ± 7.15 years (range 11–36 years). There were four chief nurses, six associate chief nurses, four supervisory nurses, two chief physicians, and two associate chief physicians. All of them held a master’s degree or above. Two rounds of expert consultation were carried out, with questionnaires sent out and collected via email. The expert familiarity (Cs) was 0.822, the judgment basis coefficient (Ca) was 0.903, and the authority coefficient (Cr) was 0.863. Expert opinions had Kendall's W coefficients of 0.208 (*P* < 0.001) and 0.242 (*P* < 0.001), respectively. During the first round of consultation, 18 expert consultation questionnaires were distributed, and all were recovered (a 100% recovery rate), with 10 experts proposing changes. The final version of the scheme was created after distributing 18 expert consultation questionnaires in the second round, achieving a recovery rate of 100.00% with no modification proposals. The discharge readiness nursing pathway based on ITHBC is shown in Figure 2.

**Figure 2:**
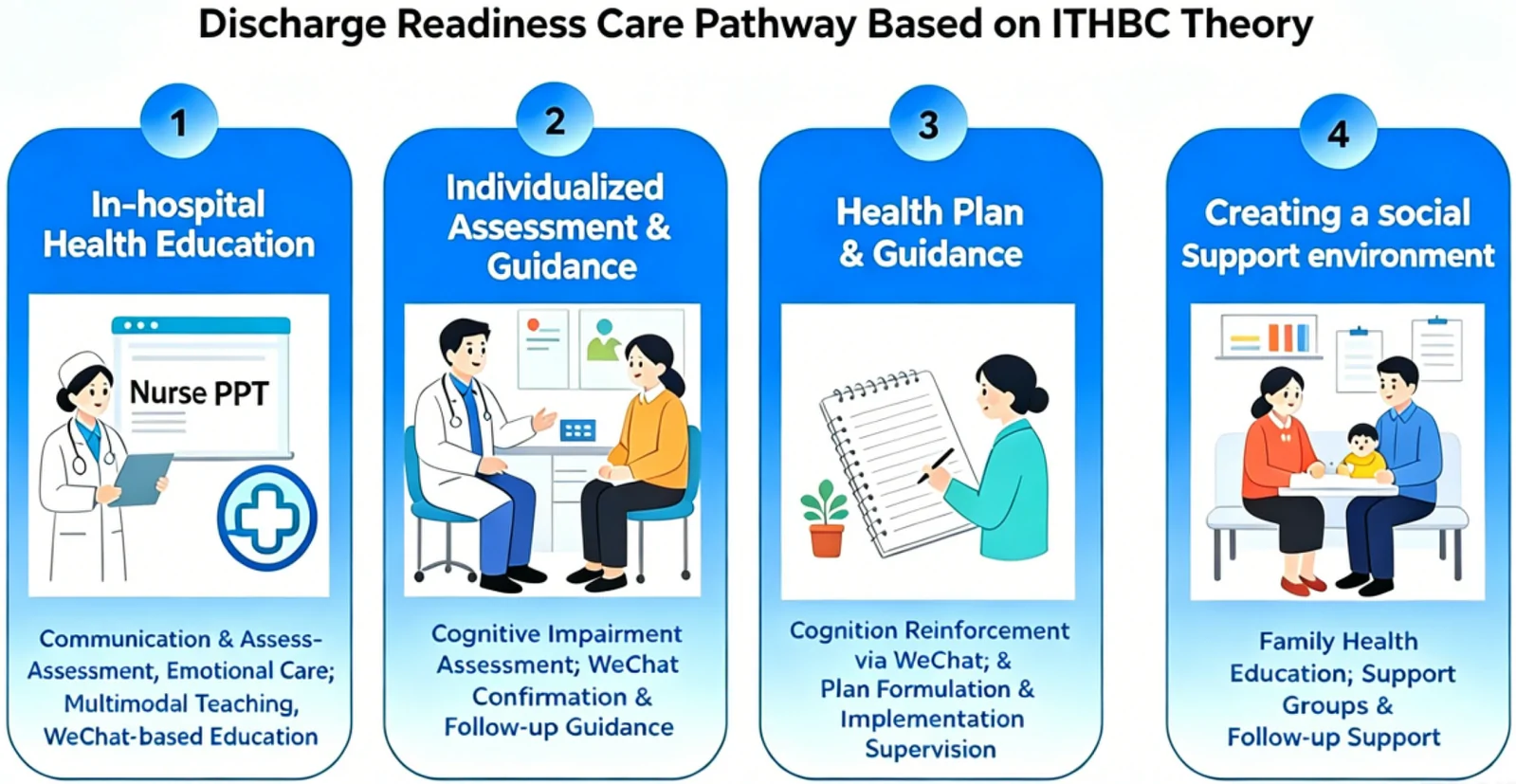
Discharge readiness care pathway based on ITHBC.

### 2.3 Intervention method

Regular health education and discharge instructions were provided to the control group. The observation group received the ITHBC-based nursing pathway from admission through 6 months after discharge, delivered by a multidisciplinary team (head nurse, neonatology nurses, neonatologist, obstetrician, rehabilitation physician, and psychologist). On the day of admission, a one-on-one session (30–45 min) was conducted. During hospitalization, individualized education and skill training were provided at least twice weekly. Within 24–48 h before discharge, a comprehensive readiness assessment and reinforced guidance were performed. After discharge, the intervention continued with weekly WeChat educational content and monthly telephone follow-ups for 6 months, plus a final outpatient review at 6 months; each contact lasted 10–20 min and addressed feeding, growth, complications, and maternal mood. The specific contents are summarized in Table 1. During the study period, no major changes occurred in unit staffing, clinical protocols, discharge criteria, or routine follow-up procedures, apart from the implementation of the ITHBC-based pathway.

**Table 1:** Comparison of intervention measures between the two groups.

Module	Control group	Observation group
In-hospital health education	Routine admission education; Large class every Tuesday (voluntary); Distribution of regular brochures; Weekly science popularization via WeChat group (synchronized with class).	One-on-one communication on admission day using a self-made education manual; Collection of baseline data; assessment with SAS and SDS self-rating anxiety scale and self-rating depression scale; Multi-form education (family care, prognosis, etc.); Face-to-face needs assessment and answers; WeChat group distribution of information and guidance on use.
Individualized assessment and guidance	Verbal inquiry about knowledge before discharge; oral supplementary guidance; No standardized assessment tool.	Assessment of cognition, feeding knowledge and skills, self-management, beliefs, psychology, home and family support; Analysis of hindering factors in the behavior stage and evaluation of effects; Confirm the use of WeChat group and guide follow-up.
Health plan and health guidance	Written discharge instruction issued at discharge; No personalized plan.	WeChat platform strengthens cognition, skills, and expert interaction; establishes beliefs and goals; Supervise self-management and evaluate feedback; Team analysis of problems; guidance and personalized behavior plans (exercise, diet, etc.).
Creating a social support environment	Family members routinely informed to participate in care; No active health education for family members; No patient exchange meeting.	Assessment of social environment and education of family members; Exchange meetings to provide emotional/informational support; Monitoring of behavior and rehabilitation; follow-up support as needed.

### 2.4 Evaluation methods

The readiness of mothers of preterm infants for hospital discharge served as the study's key outcome variable. The quality of discharge advice, care ability, psychological status, health status during the follow-up period, and nursing satisfaction were the secondary outcomes. Specific evaluation methods and tools are as follows:

#### 2.4.1 General information

Preterm infants and their mothers’ clinical and demographic information were gathered. Gestational age at birth, gender, birth weight, birth length, head circumference at birth, Apgar score at 1 min, and duration of hospital stay were included in the data for preterm newborns. Maternal data included age, gravidity, parity, mode of delivery, payment method for medical expenses, residence, cause of preterm birth, abortion history, and disease history.

#### 2.4.2 Primary outcome

Discharge Readiness: Mothers of preterm infants were evaluated for discharge readiness using the Chinese version of the Readiness for Hospital Discharge Scale-Parent Form (RHDS-Parent Form) (7). This scale is intended to assess parents of hospitalized children's preparedness for discharge. The 23 elements are divided into four categories: disease knowledge, coping skills, expected support, and personal status. With a total score ranging from 0 to 230, a higher score denotes better discharge preparation. A 10-point Likert scoring system is used (0 = totally unprepared, 10 = fully prepared). With a Cronbach's *α* coefficient of 0.85–0.90 for the scale as a whole and 0.70–0.86 for each dimension, the scale has shown strong validity and reliability.

#### 2.4.3 Secondary outcome

Quality of discharge instruction and discharge care ability: The Pediatric Quality of Discharge Instruction Scale-Parent (Ped QDTS-Parent), updated by Chen and Bai, was used to assess the quality of discharge instruction (7). This measure, which includes 18 items and two dimensions (content acquisition and guidance abilities), evaluates parents’ perceptions of the quality of nurses’ discharge counseling. The total score ranges from 0 to 180, using a 0–10 point grading system, with higher scores denoting greater discharge guidance as judged by parents. The scale's Cronbach's *α* coefficient was 0.82. The Neonatal Care Ability questionnaire developed by Xing et al. was used to evaluate the discharge care ability (8). It contains 43 items divided into three dimensions: care knowledge, care behavior, and care attitude. A 5-point Likert scoring method was utilized (0-4 points), with a total score ranging from 0 to 172 points; higher scores reflect better newborn care skills. The content validity index was 0.924, while Cronbach's *α* coefficients for the entire questionnaire and each dimension were 0.848, 0.801, 0.856, and 0.877, respectively.

Psychological status: Psychological status was evaluated at admission (within 24 h of hospitalization) and at discharge. The Chinese version of the General Self-Efficacy Scale (GSES-CV), updated by Schwarzer et al., was used to assess self-efficacy (9). The scale consists of 10 items, employs a 4-point Likert scale (1–4 points), and yields a total score ranging from 10 to 40 points. A higher score indicates a better level of general self-efficacy. In the Chinese population, the scale demonstrates strong validity and reliability. The Chinese version of the Resilience Scale for Adults (RSA-CV), updated by Yao et al., was used to assess psychological resilience (10). The redesigned scale comprises six categories with a total of 33 items: self-perception (items 1–6), future plans (items 7–10), work planning style (items 11–14), social ability (items 15–20), family harmony (items 21–26), and social resources (items 27–33). The final score ranges from 33 to 231 points using a 7-point Likert scoring system (1–7 points). A higher score indicates a greater degree of psychological resilience. The Self-Rating Anxiety Scale (SAS) (11) and the Self-Rating Depression Scale (SDS) (12) were used to measure negative feelings. An SAS score of 50–59 indicates mild anxiety, 60–69 indicates moderate anxiety, and ≥70 indicates severe anxiety, while scores below 50 are considered normal. An SDS score of 53–62 indicates mild depression, 63–72 indicates moderate depression, and ≥73 indicates severe depression; scores below 53 are considered normal. During the study period, all mothers of preterm infants underwent routine nursing admission evaluations, which included these psychological tests, and the results were documented in the nursing records.

Follow-up: Follow-up data at 6 months after discharge were obtained from the hospital's routine follow-up system (including telephone interview logs and outpatient visit records). The following variables were extracted: health status: the incidence of thrush, diaper rash, and readmission rate; growth and development indicators: daily milk intake, body length, head circumference, and body weight.

Nursing satisfaction: An assessment was conducted using a self-developed nursing satisfaction questionnaire. The Likert 5-point scoring system (5 = very satisfied, 4 = satisfied, 3 = general, 2 = dissatisfied, and 1 = extremely unhappy) was used in the questionnaire. Total satisfaction was calculated as (very satisfied + satisfied) number/total number × 100%.

### 2.5 Statistical methods

Data analysis was performed using SPSS 27.0 software. To enhance data quality, all information was extracted using standardized electronic forms, and two investigators independently cross-checked key variables before analysis. The measurement data were initially examined for homogeneity of variance and normality, and those that met these criteria were reported as mean ± standard deviation ($\bar{x}\pm s$). For intra-group comparisons, the paired *t* test was employed; for inter-group comparisons, the independent sample *t* test was utilized. Non-normal distributions were represented by the median (interquartile range) [M (Q_25_, Q_75_)], and group comparisons were made using the Mann–Whitney U test. Frequency (%) was used to convey count data, and Fisher's exact test or the chi-square test was used to compare groups.

To account for potential baseline differences between groups, analysis of covariance (ANCOVA) using multivariable linear regression was performed for the primary outcome (discharge readiness), two key secondary outcomes (discharge instruction quality and neonatal care ability), and the 6-month infant growth outcomes (daily milk intake, body length, head circumference, and body weight), with adjustments for maternal age, gestational age, birth weight, mode of delivery, payment method, and residence. Adjusted mean differences (i.e., unstandardized regression coefficients for group) with 95% confidence intervals were reported. Normality of residuals was assessed graphically using normal probability plots; no serious violations were observed. Multicollinearity was assessed using the variance inflation factor (VIF). Bar graphs were created using GraphPad Prism 10.1.2. Statistical significance was defined as *P* < 0.05. In addition, a *post hoc* power analysis was performed for the primary outcome (discharge readiness) based on the observed effect size (Cohen's d = 0.821), with a two-sided *α* of 0.05 and sample sizes of 57 and 58. The resulting statistical power was >0.99, indicating that the study had sufficient power to detect the observed difference in discharge readiness between the two groups. However, because *post hoc* power is derived from the observed effect size, this result should be interpreted with caution and does not substitute for an *a priori* sample size calculation.

## 3 Results

### 3.1 Comparison of general data

This study included 115 mothers of preterm infants, 57 of whom were in the observation group and 58 in the control group. Tables 2, 3 illustrate the general data analysis results, indicating no significant difference (*P* > 0.05) in the general data of preterm newborns and their mothers between the two groups.

**Table 2:** Comparison of general data of preterm infants [n (%), M (Q_25_, Q_75_), $\bar{x}\pm s$].

Variables	Control group (*n* = 58)	Observation group (*n* = 57)	*t/Z/χ^2^*	*P*
Gender			0.482	0.488
Male	34 (58.62)	37 (64.91)		
Female	24 (41.38)	20 (35.09)		
Gestational age	36.20 (34.43, 36.50)	35.60 (35.10, 36.30)	−1.205	0.228
Birth weight (g)	2,476.03 ± 514.34	2,488.95 ± 457.57	−0.142	0.887
Birth length (cm)	47 (43, 48)	48 (45, 48)	−0.901	0.367
Head circumference at birth (cm)	33 (32, 33)	33 (32, 33)	−0.541	0.588
Apgar score	7 (6, 10)	7 (6, 8)	−1.793	0.073
Length of stay (d)	9 (9, 9)	9 (9, 9)	−0.676	0.499

**Table 3:** Comparison of general information of mothers [n (%), M (Q_25_, Q_75_), $\bar{x}\pm s$].

Variables	Control group (*n* = 58)	Observation group (*n* = 57)	*t/Z/χ^2^/*Fisher exact test	*P*
Age (years)	27.79 ± 5.37	29.68 ± 5.08	−1.940	0.055
Number of pregnancies	2 (1, 4)	3 (1, 4)	−1.612	0.107
Order of birth	2 (1, 2)	2 (1, 3)	−0.862	0.388
Mode of delivery			0.107	0.743
Cesarean section	52 (89.66)	50 (87.72)		
Natural birth	6 (10.34)	7 (12.28)		
Payment methods for medical expenses			—	0.114^a^
Out of pocket	38 (65.52)	27 (47.37)		
Medical insurance	19 (32.76)	28 (49.12)		
Other	1 (1.72)	2 (3.51)		
Place of residence			3.529	0.060
Urban area	39 (67.24)	47 (82.46)		
County	19 (32.76)	10 (17.54)		
Causes of preterm birth			0.756	0.685
Maternal factors	27 (46.55)	22 (38.59)		
Fetal/placental factors	26 (44.83)	29 (50.88)		
Other factors	5 (8.62)	6 (10.53)		
History of miscarriage			2.516	0.113
Yes	25 (43.10)	33 (57.89)		
No	33 (56.90)	24 (42.11)		
History of disease			1.952	0.162
Yes	22 (37.93)	29 (50.88)		
No	36 (62.07)	28 (49.12)		

a is Fisher's exact test.

### 3.2 Comparison of primary outcome measures

According to the study results, mothers of preterm infants in the observation group had significantly higher RHDS-Parent Form scores than those in the control group (*P* < 0.05) (Table 4).

**Table 4:** Comparison of RHDS-parent form scores ($\bar{x}\pm s$).

Group	Number of cases	Scores
Control group	58	178.05 ± 13.45
Observation group	57	189.53 ± 14.48
Cohen’s d		0.821
95% CI		0.439–1.201
*t*		−4.404
*P*		＜0.001

### 3.3 Comparison of secondary outcome indicators

Mothers of preterm infants in the observation group had significantly higher scores on the Ped QDTS-Parent [mean difference = −18.25, 95% confidence interval (CI): −24.35 to −12.15, *P* < 0.001] and the Neonatal Care Ability Questionnaire (mean difference = −22.44, 95% CI: −29.77 to −15.12, *P* < 0.001) compared to the control group (Figure 3).

**Figure 3:**
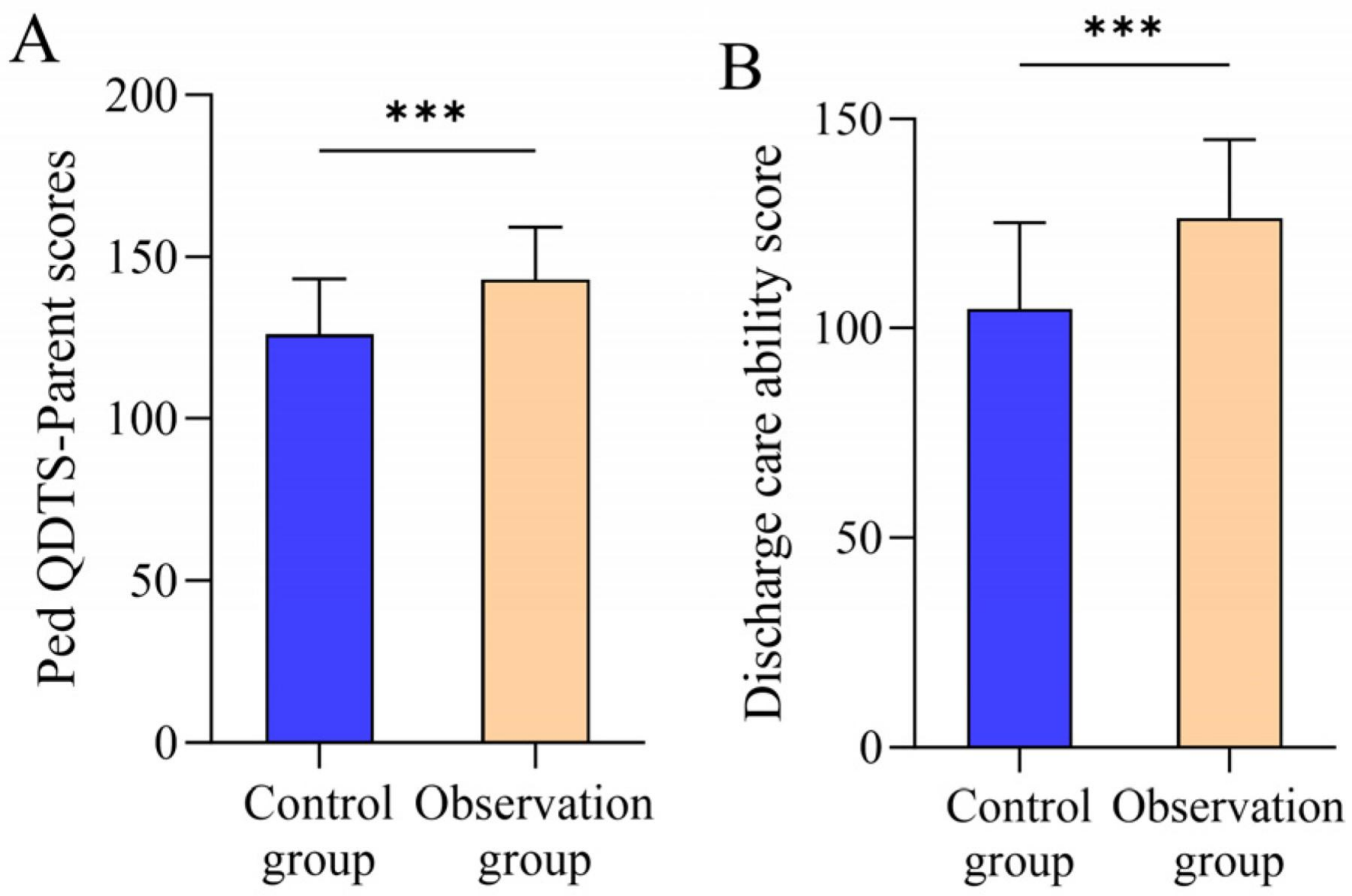
Comparison of discharge instruction quality and discharge care competence. **(A)** A comparison of the Ped QDTS-Parent scores between the two groups. **(B)** A comparison of the discharge care ability between the two groups. ***P < 0.001.

Psychological state markers did not significantly differ between the two groups at admission (all *P* > 0.05). At discharge, the observation group had significantly higher GSES-CV (mean difference = −4.53, 95% CI: −5.93 to −3.14, *P* < 0.001) and RSA-CV (mean difference = −15.19, 95% CI: −20.87 to −9.52, *P* < 0.001) scores, and significantly lower SAS (mean difference = 6.70, 95% CI: 4.75–8.66, *P* < 0.001) and SDS (mean difference = 6.59, 95% CI: 4.50–8.68, *P* < 0.001) scores compared with the control group (Figure 4). Within-group improvements from admission to discharge were also significant in both groups (all *P* < 0.05).

**Figure 4:**
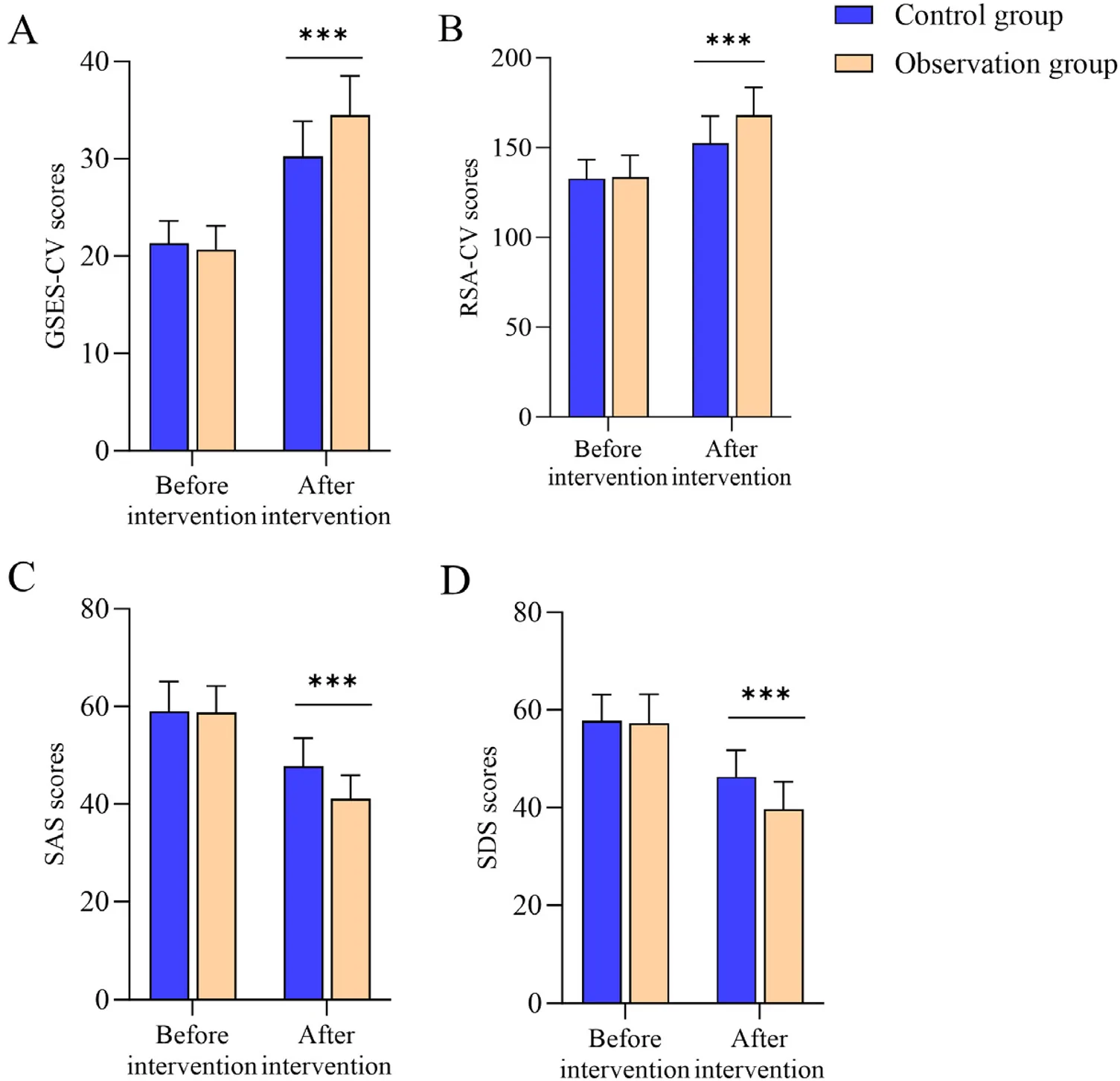
Comparison of mental states. **(A)** A comparison of the GSES-CV scores between the two groups. **(B)** A comparison of the RSA-CV scores between the two groups. **(C)** A comparison of the SAS scores between the two groups. **(D)** A comparison of the SDS scores between the two groups. ***P < 0.001.

Six months after discharge, the observation group had significantly better growth outcomes, including higher daily milk volume (mean difference = −5.55, 95% CI: −7.39 to −3.71, *P* < 0.001), body length (mean difference = −1.67, 95% CI: −2.59 to −0.76, *P* < 0.001), and body weight (mean difference = −0.32, 95% CI: −0.59 to −0.05, *P* = 0.022); head circumference also favored the observation group (*Z* = −6.335, *P* < 0.001) (Figure 5). The incidence of thrush, diaper rash, and readmission was significantly lower in the observation group (Table 5). Mothers of preterm infants in the observation group reported an overall nursing satisfaction of 96.49% (55/57), while mothers in the control group reported an overall nursing satisfaction of 77.59% (45/58). Mothers of preterm infants in the observation group reported significantly greater nursing satisfaction than those in the control group (*χ^2^* = 9.059, *P* = 0.003).

**Table 5:** Comparison of health status between the two groups [n (%)].

Group	Thrush	Diaper rash	Readmission rate
Control group (*n* = 58)	8 (13.79)	13 (22.41)	10 (17.24)
Observation group (*n* = 57)	1 (1.75)	4 (7.02)	2 (3.51)
*χ^2^*/Fisher exact test	—	5.409	5.801
*P*	0.032^a^	0.020	0.016

a Fisher's exact test.

**Figure 5:**
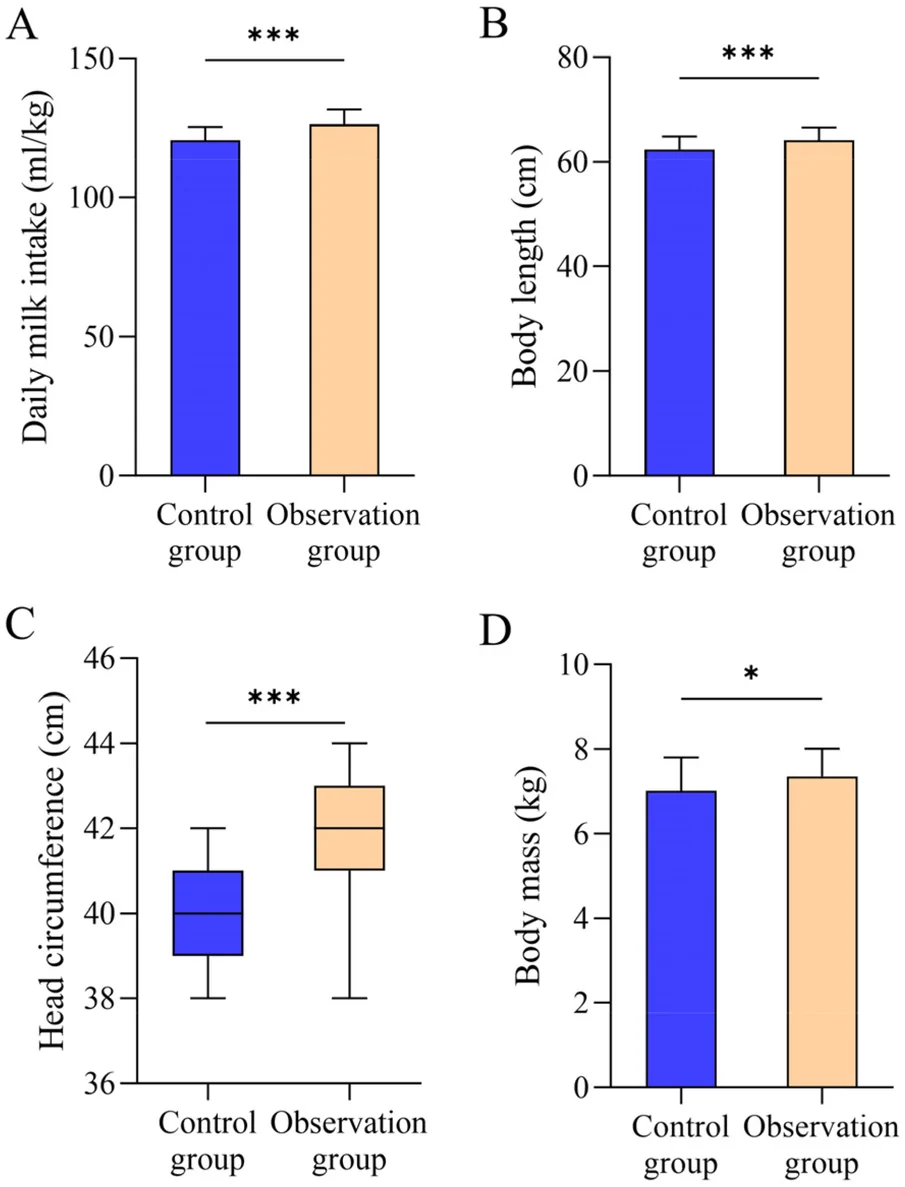
Comparison of growth and development indicators. **(A)** A comparison of the daily milk intake between the two groups. **(B)** A comparison of the body length comparison between the two groups. **(C)** A comparison of the head circumference between the two groups. **(D)** A comparison of the body weight between the two groups. ***P < 0.001; *P < 0.05.

### 3.4 Covariate-adjusted analyses for key outcomes

To further account for potential baseline differences, analysis of covariance using multivariable linear regression was conducted for the primary outcome (discharge readiness), two key secondary outcomes (discharge instruction quality and neonatal care ability), and the 6-month infant growth outcomes (daily milk intake, body length, head circumference, and body weight), with adjustment for maternal age, gestational age, birth weight, mode of delivery, payment method, and residence. After adjustment, the observation group remained significantly superior to the control group on all these outcomes (all *P* < 0.05). The adjusted mean differences and 95% confidence intervals are summarized in Table 6. Collinearity diagnostics showed no serious multicollinearity (all VIF < 2).

**Table 6:** Adjusted differences between the two groups for key outcomes.

Outcome	Adjusted mean difference (observation − control)	95% CI	*P*
Discharge readiness	10.81	5.29–16.32	<0.001
Discharge instruction quality	17.07	10.59–23.55	<0.001
Neonatal care ability	22.01	14.29–29.73	<0.001
Daily milk intake	5.33	3.46–7.20	<0.001
Body length	1.71	0.73–2.68	<0.001
Head circumference	1.58	1.11–2.04	<0.001
Body weight	0.29	0.01–0.57	0.043

Adjusted for maternal age, gestational age, birth weight, mode of delivery, payment method, and residence.

## 4 Discussion

For families of preterm infants, the move from the neonatal intensive care unit (NICU) to the home is a crucial and delicate time. As soon as they are discharged, mothers of preterm infants are required to perform demanding care responsibilities. But many women feel unprepared for these responsibilities, which could harm the health of their preterm infants and raise the possibility of unscheduled readmissions (13, 14). Although discharge readiness as a key factor affecting the level of post-discharge adjustment has been gradually paid attention to, traditional discharge guidance is often generalized in content and concentrated in time, which is difficult to fully respond to the dynamic psychological and behavioral needs of mothers (15). ITHBC provides a structured framework for solving such shortcomings. The theory emphasizes that the formation of health behavior needs to go through a gradual process of knowledge acquisition, belief strengthening and self-efficacy improvement, and relies on the continuous promotion of social support (6). Based on ITHBC, a thorough nursing pathway was developed and assessed in this study. The findings suggest that the pathway may be associated with better discharge readiness, discharge advice quality, care ability, and mental health among mothers of preterm infants, as well as improved health status of preterm infants upon discharge.

The overall effect of the ITHBC-based nursing pathway may be largely attributable to its multi-stage and individualized design, which integrates information supply, skill training, psychosocial support, and environment optimization (16). In contrast to the traditional practice of focusing education on the point of discharge, this care pathway was implemented starting at the time of maternal admission and continued throughout the entire cycle of hospitalization, discharge preparation, and post-discharge follow-up. This longitudinal engagement process is coordinated by a multidisciplinary team, which can repeatedly assess mothers’ needs and stages of behavior change, and implement targeted strategies to match the stage, which is crucial for the internalization and maintenance of healthy behaviors (6, 17). From an implementation perspective, the intervention was delivered by an established multidisciplinary team within routine clinical workflows, which likely contributed to its feasibility. However, potential barriers such as nurses’ workload, maternal engagement with the WeChat platform, and variability in delivery fidelity were not formally measured. Future studies should assess implementation process indicators to better understand how the pathway can be sustained and scaled.

The study's findings showed that mothers in the observation group had a significantly higher preparedness for hospital discharge score than those in the control group (*P* < 0.05). The RHDS scale takes into account a number of factors, including expected support, coping skills, disease awareness, and personal status. The ITHBC nursing pathway gave targeted intervention to these dimensions. The use of individualized health education and self-compiled manuals may have contributed to a significant improvement in mothers’ awareness of preterm infant care, potential complications, and follow-up plans (18). At the same time, continuous evaluation of the psychological state of mothers and targeted emotional nursing may have helped to improve their personal state and perceived coping ability (19). The establishment of a WeChat group and “preterm maternal exchange meeting” may have created a supportive community atmosphere and strengthened anticipatory social support (20). The aforementioned results are in line with those of Tiryaki et al. (2), who demonstrated in a randomized controlled trial that the family integrated care model can greatly enhance parental discharge preparedness. Although our quasi-experimental design provides less robust causal evidence, the consistency of findings is noteworthy. The ITHBC approach of this study further emphasized support within a theory-driven behavioral change framework to ensure that readiness is not only informational, but also psychological and behavioral.

Mothers in the observation group also perceived higher quality of discharge guidance and showed stronger neonatal care ability. The ITHBC pathway clearly includes a comprehensive assessment of maternal care cognition, skills, home environment, and family support before discharge, so that a multidisciplinary team can identify and make up for individualized deficiencies before discharge. Meanwhile, the WeChat platform was used to strengthen skills, promote expert interaction, and guide mothers to set and achieve personalized behavioral goals (21). This process-based approach, which emphasizes exercises, feedback, and self-monitoring, has been suggested to be more effective than passive information transfer in improving care competence (22). Research has demonstrated that a key predictor of parents’ preparedness and post-discharge coping challenges is the quality of discharge instruction (23). The findings of this study also suggested that the theory-based interactive education model might enhance mothers’ actual caring skills as well as their perception of the quality of instruction.

Maternal mental health is a critical yet often overlooked aspect of discharge preparation (24). This study showed that maternal self-efficacy, resilience, anxiety, and depression improved over time in both groups, reflecting the general benefits of structured NICU support services. On all psychological measures, however, the observation group outperformed the control group by a substantial margin (*P* < 0.05). Since admission, the ITHBC pathway has used SAS and SDS for routine psychological assessment, which can detect emotional discomfort early and provide focused emotional nursing. Simultaneously, the improvement of self-efficacy is not only the fundamental construct of ITHBC, but also the confirmed mediator variable between discharge readiness and after-discharge coping issues of mothers of preterm infants (5). Through the provision of success experiences through guided skill practice, vicariously learning through peer support groups, and verbal persuasion through continued professional encouragement, the intervention most likely enhanced maternal self-efficacy and subsequently alleviated symptoms of anxiety and depression (25, 26). Furthermore, the cultivation of social resources and family cohesion through the “creating a social support environment” aspect may also have promoted the improvement of psychological resilience (27). This is consistent with the results of Duran et al. (4), which demonstrated the robust correlation between infant care practices and maternal mental health. Consistent with previous studies, enhancing maternal self-efficacy and providing social support appear to be particularly important mechanisms underlying improved discharge readiness (19, 20). However, because the intervention was delivered as a multi-component package, the independent contribution of each element could not be determined in this study. Overall, the data from this study suggest that an integrated intervention targeting both care knowledge and maternal emotional wellbeing may produce synergistic benefits.

At 6 months after discharge, the observation group showed more favorable infant outcomes, including greater daily milk volume, body length, head circumference, and weight, as well as lower rates of thrush, diaper rash, and readmission. These findings may reflect the long-term benefits of maternal empowerment and increased awareness of health surveillance. Prepared mothers may be better able to recognize early warning signs, follow feeding and hygiene practices, and seek prompt healthcare, which may help prevent common complications and reduce avoidable readmissions (15). This finding echoes the results of the TRAMPRE trial, which employed a randomized controlled design and confirmed the positive impact of discharge training based on the transition period model on post-discharge outcomes in late preterm infants (28). Although our study used a non-randomized design, the comparable direction of effects supports the potential benefit of structured discharge planning. Both studies highlight the important value of structured, theory-based discharge planning in bridging NICU discharge with home care. However, these differences could also have been influenced by factors other than the intervention, such as feeding practices or access to follow-up care, and should therefore be interpreted cautiously. Despite these caveats, the ITHBC-based approach in this study, with its emphasis on continuous behavior maintenance and social support, may offer additional advantages in promoting long-term adherence to recommended care behaviors. The higher nursing satisfaction of mothers in the observation group further supported the acceptability and value identification of this comprehensive care model.

This study has several limitations. The single-center design, relatively small sample size (determined pragmatically without an *a priori* power calculation), and use of a historical (temporal) control rather than concurrent enrollment limit generalizability and may introduce selection bias and confounding by secular changes. Temporal separation may have altered participant characteristics and contextual factors (e.g., referral patterns, case mix, disease spectrum, hospital policies, staffing, clinical protocols, or discharge criteria), and the unequal enrollment windows (9 vs. 5 months) may have introduced seasonal variation. Although no major changes occurred during the study period and baseline characteristics were comparable, multivariable adjustment can only partially account for these differences, and residual time-related confounding may persist. The ITHBC-based pathway combined multiple components, so the observed effects cannot be attributed to any single element or to the theory itself, and the design could not isolate their individual contributions. Subjective scale ratings may be affected by social expectation bias, and the nursing satisfaction questionnaire has not been formally validated. The follow-up was limited to 6 months, and 6-month infant outcomes may have been influenced by unmeasured factors such as feeding type, breastfeeding rates, socioeconomic status, and distance to follow-up care. Binary outcomes and some psychological measures were not adjusted because of small event counts or baseline comparability. Future studies should adopt multicenter, large-sample randomized controlled designs with extended follow-up and blinded evaluation of objective outcomes.

## 5 Conclusion

The ITHBC-based nursing pathway was associated with significant improvements in discharge readiness, discharge guidance quality, care ability, and mental health among mothers of preterm infants, as well as more favorable infant health outcomes at 6 months after discharge. This comprehensive intervention, which has a theoretical foundation, interdisciplinary cooperation, and technical support, is a workable and successful way to improve ongoing care for preterm infants and their families. It also serves as a useful guide for improving routine discharge services for preterm infants.

## Data Availability

The original contributions presented in the study are included in the article/Supplementary Material, further inquiries can be directed to the corresponding author.
